# Chromosome-level genome assembly of *Hydractinia symbiolongicarpus*

**DOI:** 10.1093/g3journal/jkad107

**Published:** 2023-05-18

**Authors:** Koto Kon-Nanjo, Tetsuo Kon, Helen R Horkan, Febrimarsa, Robert E Steele, Paulyn Cartwright, Uri Frank, Oleg Simakov

**Affiliations:** Department of Neurosciences and Developmental Biology, University of Vienna, Vienna 1030, Austria; Department of Neurosciences and Developmental Biology, University of Vienna, Vienna 1030, Austria; Centre for Chromosome Biology, School of Biological and Chemical Sciences, University of Galway, Galway H91W2TY, Ireland; Centre for Chromosome Biology, School of Biological and Chemical Sciences, University of Galway, Galway H91W2TY, Ireland; Department of Biological Chemistry, University of California Irvine, Irvine, CA 92697-1700, USA; Department of Ecology and Evolutionary Biology, University of Kansas, Lawrence, KS 66045, USA; Centre for Chromosome Biology, School of Biological and Chemical Sciences, University of Galway, Galway H91W2TY, Ireland; Department of Neurosciences and Developmental Biology, University of Vienna, Vienna 1030, Austria

**Keywords:** *Hydractinia*, chromosome-level genome assembly, HSymV2.0, stem cell, hydrozoa

## Abstract

*Hydractinia symbiolongicarpus* is a pioneering model organism for stem cell biology, being one of only a few animals with adult pluripotent stem cells (known as i-cells). However, the unavailability of a chromosome-level genome assembly has hindered a comprehensive understanding of global gene regulatory mechanisms underlying the function and evolution of i-cells. Here, we report the first chromosome-level genome assembly of *H. symbiolongicarpus* (HSymV2.0) using PacBio HiFi long-read sequencing and Hi-C scaffolding. The final assembly is 483 Mb in total length with 15 chromosomes representing 99.8% of the assembly. Repetitive sequences were found to account for 296 Mb (61%) of the total genome; we provide evidence for at least two periods of repeat expansion in the past. A total of 25,825 protein-coding genes were predicted in this assembly, which include 93.1% of the metazoan Benchmarking Universal Single-Copy Orthologs (BUSCO) gene set. 92.8% (23,971 genes) of the predicted proteins were functionally annotated. The *H. symbiolongicarpus* genome showed a high degree of macrosynteny conservation with the *Hydra vulgaris* genome. This chromosome-level genome assembly of *H. symbiolongicarpus* will be an invaluable resource for the research community that enhances broad biological studies on this unique model organism.

## Introduction

*Hydractinia symbiolongicarpus* is a colonial hydrozoan cnidarian species. The organism is distributed along the east coast of North America from Maine to South Carolina (Buss and Yund 1989). In its natural habitat, *H. symbiolongicarpus* forms colonies on hermit crab-occupied gastropod shells. In the laboratory, *H. symbiolongicarpus* can also form colonies on glass slides and be cultured in artificial seawater. A number of laboratory strains are available for this species. Colonies have functionally specialized zooids (polyps), including the feeding polyp (gastrozooid), reproductive polyp (gonozooid), and defensive polyp (dactylozooid), which are connected by the stolonal tissue, a shared gastrovascular system (Frank *et al*. 2020). While forming clonal colonies, *H. symbiolongicarpus* also spawns gametes daily, providing convenient access to embryogenesis. This trait facilitates gene expression manipulation using transient RNAi-mediated genetic knockdown (DuBuc *et al*. 2020; Quiroga-Artigas *et al*. 2020), CRISPR/Cas9-mediated genome editing (Gahan *et al*. 2016; Sanders *et al*. 2018), and random-integration transgenesis (Künzel *et al*. 2010). Various transgenic animal clones are currently available (Chrysostomou *et al*. 2022).

*Hydractinia* is the first animal in which stem and germ cells were described by August Weismann in the 19th century (Weismann 1883). The animal has a unique population of adult pluripotent stem cells, known as interstitial stem cells (i-cells for short). i-cells continuously produce progenitors to all somatic and germ cell lineages (DuBuc *et al*. 2020; Varley *et al*. 2023). A single i-cell grafted from one colony to another can self-renew and differentiate into all somatic lineages and germ cells (Varley *et al*. 2023). The pluripotency of *H. symbiolongicarpus* i-cells contrasts with the multipotency of *Hydra vulgaris* i-cells, which produce the neuroglandular lineage and germ cells but not epidermal and gastrodermal epithelial cells (Bosch and David 1987; Bode 1996; Hobmayer *et al*. 2012; Juliano *et al*. 2014). *H. symbiolongicarpus* is an excellent model animal for studying the function and evolution of stem cells in multicellular organisms (Plickert *et al*. 2012).

The genome size of *H. symbiolongicarpus* was estimated to be 514 Mb (Frank *et al*. 2020). *H. symbiolongicarpus* has 15 chromosomes revealed by the G-banding method (Chen *et al*. 2023). This chromosome number is similar in other cnidarians such as *H. vulgaris* (Simakov *et al*. 2022; Cazet *et al*. 2023). In addition, there are genetic linkage maps available, which are composed of 15 linkage groups (Chen *et al*. 2023). So far, the available genome sequence of *H. symbiolongicarpus* is limited to a contig-level (>4,800 contigs) assembly called Hsym_primary_v1.0 (https://research.nhgri.nih.gov/hydractinia/). The unavailability of a chromosome-level genome assembly has hindered investigation into many aspects of stem cell function in *Hydractinia*, such as promoter–enhancer interactions. As such, production of a chromosome-level genome assembly of *H. symbiolongicarpus* has been anticipated for a long time by the scientific community and will provide a comprehensive landscape of the genome.

Here, we report the first chromosome-level genome assembly of *H. symbiolongicarpus* (HSymV2.0) using PacBio high-fidelity (HiFi) sequencing and Hi-C scaffolding. The final assembly is 483 Mb in total length with 15 chromosomes, representing 99.8% of the total assembly size. It will be an invaluable resource for studying molecular regulatory mechanisms underlying development, stem cells, regeneration, and their evolution.

## Materials and methods

### Animal husbandry

Colonies of *H. symbiolongicarpus* were maintained as described previously (Frank *et al*. 2020). The animals were grown on glass slides kept in artificial seawater at 20–22°C. The animals were kept in a constant 14:10 light:dark cycle.

### DNA sequencing library preparation and sequencing

High-molecular weight genomic DNA was extracted from male feeding polyps (clone 291-10) of *H. symbiolongicarpus*. A PacBio HiFi library was prepared according to the manufacturer's protocol and sequenced using the PacBio Sequel II platform. For Hi-C library preparation, male (clone 291-10) feeding polyps of *H. symbiolongicarpus* were flash frozen and ground. Following the manufacturer's instructions, a Hi-C library was prepared using the Dovetail Omni-C kit (Canta Bio, #21005). The insert size distribution of the Hi-C library was confirmed to be approximately between 350 bp and 1,000 bp using Agilent Fragment Analyzer (Agilent). The Hi-C library was sequenced with 150 bp paired-end reads using Illumina MiSeq by the Next Generation Sequencing core facility of the Vienna BioCenter.

### Genome assembly and scaffolding

Statistics of the PacBio HiFi read quality were calculated using NanoPlot v1.41.0 (De Coster *et al*. 2018). PacBio HiFi reads were assembled into contig sequences using Hifiasm v0.16.1-r375 (Cheng *et al*. 2021) with default parameters. The chromosome-level scaffolds were constructed with the combination of the contig sequences and the Hi-C sequencing reads. The Hi-C reads were aligned to the contig sequences using Juicer v1.6 (Durand *et al*. 2016b) with default parameters. Then, Hi-C scaffolding was performed using the 3D-DNA pipeline (Dudchenko *et al*. 2017) with parameters “–editor-repeat-coverage 1000 -r 0.” Scaffolds were manually curated using Juicebox Assembly Tools v1.11.08 (Durand *et al*. 2016a). The final assembly was named HSymV2.0. Assembly quality and completeness were evaluated using QUAST v5.2.0 (Gurevich *et al*. 2013) and Benchmarking Universal Single-Copy Orthologs (BUSCO) v5.2.2 (Simão *et al*. 2015) with the metazoa odb10 database (metazoa_odb10).

### Validation of the genome assembly with *H. symbiolongicarpus* linkage maps

In order to anchor the contig sequences of the Hsym_primary_v1.0 assembly to the previously reported genetic linkage maps of *H. symbiolongicarpus* (Chen *et al*. 2023), we merged the contig sequences based on the SNP marker positions and constructed 15 pseudochromosome sequences for the maternal and paternal genetic linkage map. These pseudochromosome sequences were aligned to the 15 chromosomal-level scaffolds of the HSymV2.0 assembly using minimap2 v2.24 (Li 2018) with the parameters “-ax asm5 –cs –secondary=no.” Alignments with mapping quality 60 were retained using samtools view (Danecek *et al*. 2021). The alignment files in the BAM format were converted to the PAF format using paftools from minimap2 v2.24. Finally, the alignments were visualized in dot plots using D-GENIES (Cabanettes and Klopp 2018).

### Identification of repetitive sequences

A custom repeat library of the genome assembly was generated using RepeatModeler v2.0.4 (Flynn *et al*. 2020) with default parameters. Next, repetitive sequences were identified using RepeatMasker v4.1.4 (http://www.repeatmasker.org) and the custom repeat library. A repeat-masked sequence of the HSymV2.0 assembly was also generated at this step. Finally, the output of RepeatMasker was summarized using the helper scripts from RepeatMasker v4.1.4. For comparison, repetitive sequences in the *H. vulgaris* strain AEP (Cazet *et al*. 2023) were identified using the same pipeline.

### Genome annotation

The repeat-masked genome assembly of the HSymV2.0 assembly was used for gene predictions. We identified the protein-coding region and untranslated region (UTR) for each gene, with a combination of ab initio prediction, homology-based prediction, and transcriptome-based prediction using BRAKER2 (Brůna *et al*. 2021). For homology-based prediction, we obtained the genome sequence and annotation of *H. vulgaris* strain 105 (GCA_022113875.1) from the National Center for Biotechnology Information (NCBI) Genome database; the genome sequence and annotation of *H. vulgaris* strain AEP (Cazet *et al*. 2023) from the Hydra AEP Genome Project Portal (https://research.nhgri.nih.gov/HydraAEP/); and the genome sequence and annotation from the contig-level reference assembly of *H. symbiolongicarpus* (Hsym_primary_v1.0) from the Hydractinia Genome Project Portal (https://research.nhgri.nih.gov/hydractinia/). Protein sequences were generated from the reference genome sequences and the genome annotations using GffRead (Pertea and Pertea 2020) with the parameter “-S -J.” For each gene, the longest isoform was extracted for homology-based prediction. Then, all protein sequences were merged. BRAKER2 was run on the repeat-masked genome assembly using the homologous protein sequences with parameters “–epmode –softmasking.” For transcriptome-based prediction, we used publicly available RNA-seq reads (accession numbers: SRR1796501–SRR1796515, SRR9331388–SRR9331403, and SRR14265606–SRR14265626, SRR18686538–SRR18686548). The repeat-masked genome assembly was indexed using STAR v2.7.10b (Dobin *et al*. 2013) with the parameter “–runMode genomeGenerate.” The RNA-seq reads were aligned to the indexed genome assembly using STAR v2.7.10b with parameters “–runMode alignReads –outSAMtype BAM SortedByCoordinate.” All alignment files were merged using samtools merge (Danecek *et al*. 2021). Then, BRAKER2 was run on the repeat-masked genome assembly using the merged bam file with parameters “–softmasking.” UTR regions were added to the transcriptome-based predictions using BRAKER2 with parameters “–softmasking –addUTR=on.” From the initial gene model predictions, consensus gene models were produced using TSEBRA v1.0.3 (Gabriel *et al*. 2021). We also transferred the gene annotation gene coordinates from the Hsym_primary_v1.0 assembly to the HSymV2.0 assembly using the Liftoff tool (Shumate and Salzberg 2020). We selected gene models that contained start and stop codons, while excluding those that had internal stop codons. Finally, gene model predictions from BRAKER2 and gene models from Liftoff were integrated using BEDTools (Quinlan and Hall 2010). To validate the completeness of the final gene models, protein sequences were generated from the HSymV2.0 assembly and the genome annotations using GffRead (Pertea and Pertea 2020). Each protein sequence was examined for the presence of homologous sequences in the NCBI-nr database using the BLAST+ v2.13.0 with the parameters “-evalue 1e-05.” Protein domain prediction was performed using InterProScan v5.60-92.0 (Zdobnov and Apweiler 2001). Gene functional annotation was performed using eggNOG-Mapper v2.1.9 (Huerta-Cepas *et al*. 2019). Mitochondrial genome annotation was performed using the MITOS WebServer (Bernt *et al*. 2013). For visualization of the mitochondrial genome annotation, a Genbank file was generated from the mitochondrial genome sequence and its annotation using the EMBOSS Seqret tool (Rice *et al*. 2000). The mitochondrial genome map was generated from the Genbank file using ApE (Davis and Jorgensen 2022).

### Synteny analysis

To identify orthologs between *H. symbiolongicarpus* and *H. vulgaris*, the longest protein isoforms from the chromosome-level scaffolds were prepared for both species. Both gene sets were compared reciprocally using blastp with default parameters (Altschul *et al*. 1990). Gene pairs with reciprocal best hits were identified as orthologs between the two species. For each species, each ortholog was numbered according to its genomic coordinate. Oxford plots were plotted using the plot function in R v4.2.2.

## Results and discussion

### Chromosome-level genome assembly of *H. symbiolongicarpus* (HSymV2.0)

**Fig. 1.**
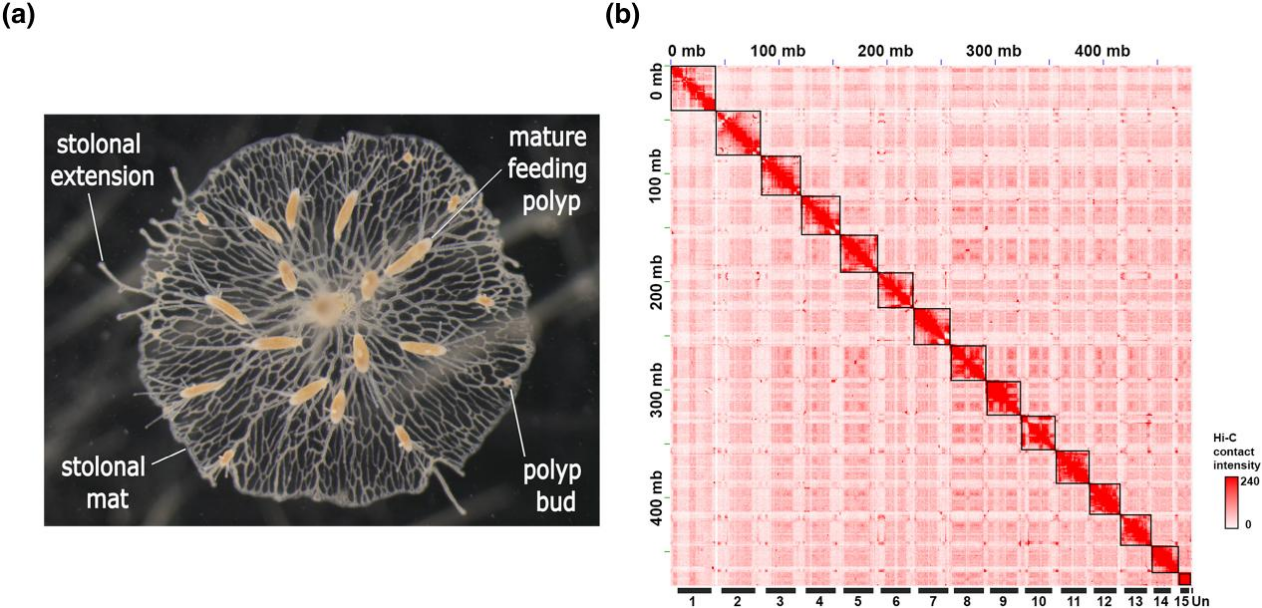
Chromosome-level genome assembly of *H. symbiolongicarpus* (HSymV2.0). a) Colony of *H. symbiolongicarpus* male (Clone 291-10) attached to a glass microscope slide, showing mature feeding polyps (∼5 mm height), polyp buds, and shared stolon network (sexual polyps not pictured as they develop later). b) Hi-C contact map showing the 15 chromosome-level scaffolds and the unplaced sequences (Un). Scaffolds are ordered by length and assigned numbers.

High-molecular weight genomic DNA was extracted from male (clone 291-10) colonies of *H. symbiolongicarpus* (Fig. 1a and Supplementary Fig. 1a), the same genetic individual from which the first version of the genome was prepared. A total of 34.0 Gb HiFi reads (66×) of the *H. symbiolongicarpus* genome were generated with a read N50 length of 13.2 kb (Table 1 and Supplementary Fig. 1b). We performed de novo genome assembly of the *H. symbiolongicarpus* genome using Hifiasm (Cheng *et al*. 2021). The initial genome assembly contains 325 contigs with a total length of 483 Mb (Table 2). The total contig length is comparable to the previously estimated genome size (Frank *et al*. 2020). The contig N50 was 28.8 Mb and the contig L50 was 8 (Table 2). The maximum contig length was 42.6 Mb (Table 2). The GC content was 35.85% (Table 2). We also assembled a 15,471 bp mitochondrial DNA scaffold which contains 13 proteins, small and large subunit rRNAs, and methionine and tryptophan tRNAs like other cnidaria species (Supplementary Fig. 2) (Zou *et al*. 2012; Lin *et al*. 2019). We evaluated the completeness of the genome assembly by searching for sets of BUSCO in the contig sequences (Simão *et al*. 2015). We used the metazoa odb10 database (metazoa_odb10) for this analysis. We identified 818 complete BUSCO genes out of 954 BUSCO genes (85.7%), of which 756 were present as single copies (79.2%) and 62 as duplicates (6.5%). We also identified 64 fragmented BUSCO genes (6.7%). Of note, these statistics of the contig sequences are better than those of the Hsym_primary_v1.0 assembly, the previously reported contig-level assembly (Table 3). For example, contig N50 of the current assembly is 13 times higher than that of Hsym_primary_v1.0 (28.8 Mb vs 2.2 Mb), and the number of contigs is less than a tenth of that of Hsym_primary_v1.0 (325 vs 4,840, Table 2). To construct a chromosome-level genome assembly, 14,836,376 Hi-C read pairs were generated from male (clone 291-10) feeding polyps (Supplementary Fig. 3). Hi-C scaffolding generated the final assembly of 483 Mb in length with 15 chromosomes which represent 99.8% of the total assembly size (Fig. 1b and Supplementary Fig. 4). The scaffold N50 was 33 Mb. The size of the largest scaffold was 42.6 Mb. The total size of the 69 unplaced sequences was 1.1 Mb. Four of the 15 scaffolds (scaffold 8, 11, 12, and 15) consist of single contigs (Supplementary Fig. 4). The Hi-C contact map of the chromosome-level genome assembly shows a low number of trans-chromosomal interactions in our genome assembly (Fig. 1b). In each chromosome, the Hi-C contact intensity at the diagonal position is stronger than that of the other position. This indicates that adjacent sequences of our assembly have strong contact. These findings suggest the completeness of the chromosome-level genome assembly. The final chromosome-level genome assembly was named HSymV2.0.

**Table 1.** Summary of the genome sequencing.

Experiment	Sequencing platform	Number of reads	Total base (base)	Read length (bp)
Whole genome sequencing	PacBio Sequel II	3,049,931	34,014,052,488	13,241 (N50)
Hi-C	Illumina MiSeq	14,836,376	4,450,912,800	150 × 2

**Table 2.** Assembly statistics of the *H. symbiolongicarpus* genome.

	Contigs	All Hi-C scaffolds (HSymV2.0)	Chromosome-level scaffolds	Hsym_primary_v1.0
Total length (bp)	482,771,323	482,928,323	481,835,061	406,663,980
Total sequence number	325	84	15	4,840
Maximum sequence length (bp)	42,595,244	42,572,105	42,572,105	11,665,964
N50 (bp)	28,808,060	33,314,500	33,314,500	2,235,630
L50	8	7	7	48
GC content (%)	35.85	35.85	35.85	35.22

**Table 3.** BUSCO scores from the genome assembly.

	Current study (HSymV2.0)	Hsym_primary_v1.0
Complete BUSCOs (C)	818 (85.7%)	796 (83.5%)
Complete and single-copy BUSCOs (S)	756 (79.2%)	722 (75.7%)
Complete and duplicated BUSCOs (D)	62 (6.5%)	74 (7.8%)
Fragmented BUSCOs (F)	64 (6.7%)	69 (7.2%)
Missing BUSCOs (M)	72 (7.6%)	89 (9.3%)
Total BUSCO groups searched	954	954

### Comparing HSymV2.0 with the preexisting genetic linkage maps and sex chromosome identification

**Fig. 2.**
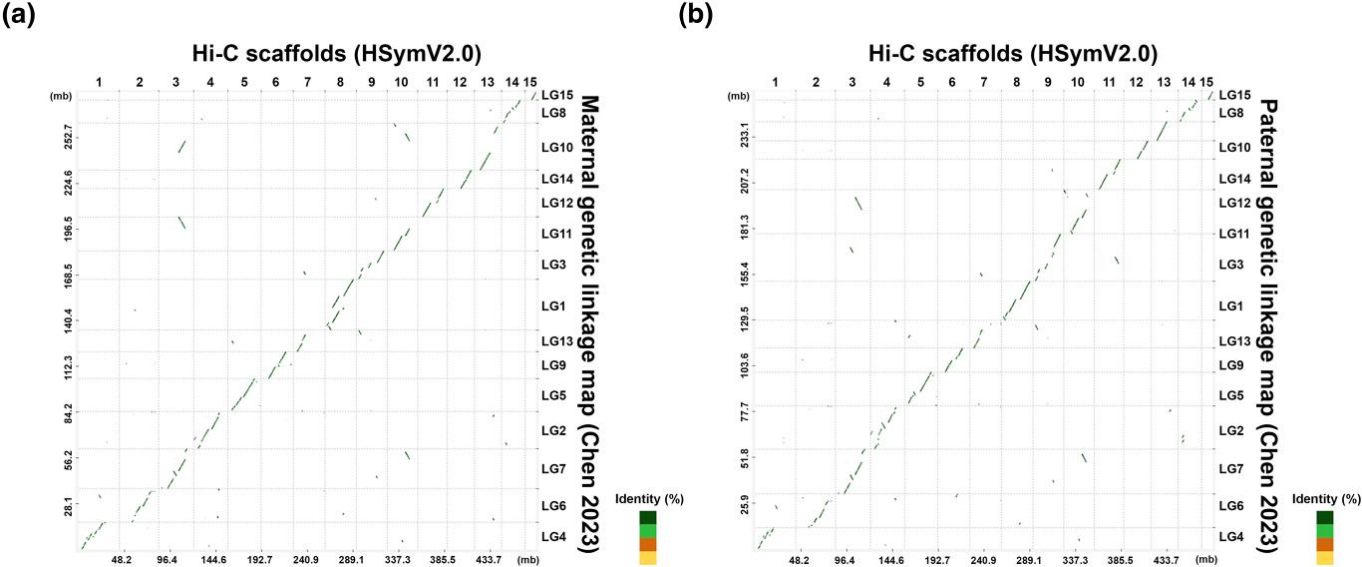
Collinearity between the Hi-C scaffolds of the present study and the genetic linkage maps (Chen *et al*. 2023). a) Sequence alignments between the Hi-C scaffolds and pseudochromosomes based on the maternal genetic linkage map. b) Sequence alignments between the Hi-C scaffolds and pseudochromosomes based on the paternal genetic linkage map.

In order to further evaluate the completeness of the HSymV2.0 assembly, we compared our genome assembly with the previously reported genetic linkage maps of *H. symbiolongicarpus* (Chen *et al*. 2023). Based on the loci of the SNP markers in the Hsym_primary_v1.0 assembly, we anchored the contig sequences of the Hsym_primary_v1.0 to the maternal and paternal genetic linkage maps. We obtained the maternal and paternal 15 pseudochromosome sequences. Then, we aligned the maternal and paternal pseudochromosomes to our 15 chromosome-level scaffolds. Both sets of 15 pseudochromosomes were uniquely mapped to our 15 chromosome-level scaffolds (Fig. 2a and b). The resulting alignments also revealed the colinear relationships between the 15 chromosome-level scaffolds of the HSymV2.0 assembly and the maternal pseudochromosomes (Fig. 2a) or paternal pseudochromosomes (Fig. 2b). These results demonstrate the contiguity and completeness of the HSymV2.0 assembly. The previous study showed that *H. symbiolongicarpus* has an XX/XY sex determination system and that the sex determining locus is located at the end of linkage group 4 (Chen *et al*. 2023). In both linkage groups, linkage group 4 was mapped to chromosome 1 of the HSymV2.0 assembly (Fig. 2a and b). Based on this result, the sex chromosome in the HSymV2.0 assembly is most likely to be chromosome 1 (Fig. 2).

### Identification of repetitive sequences

**Fig. 3.**
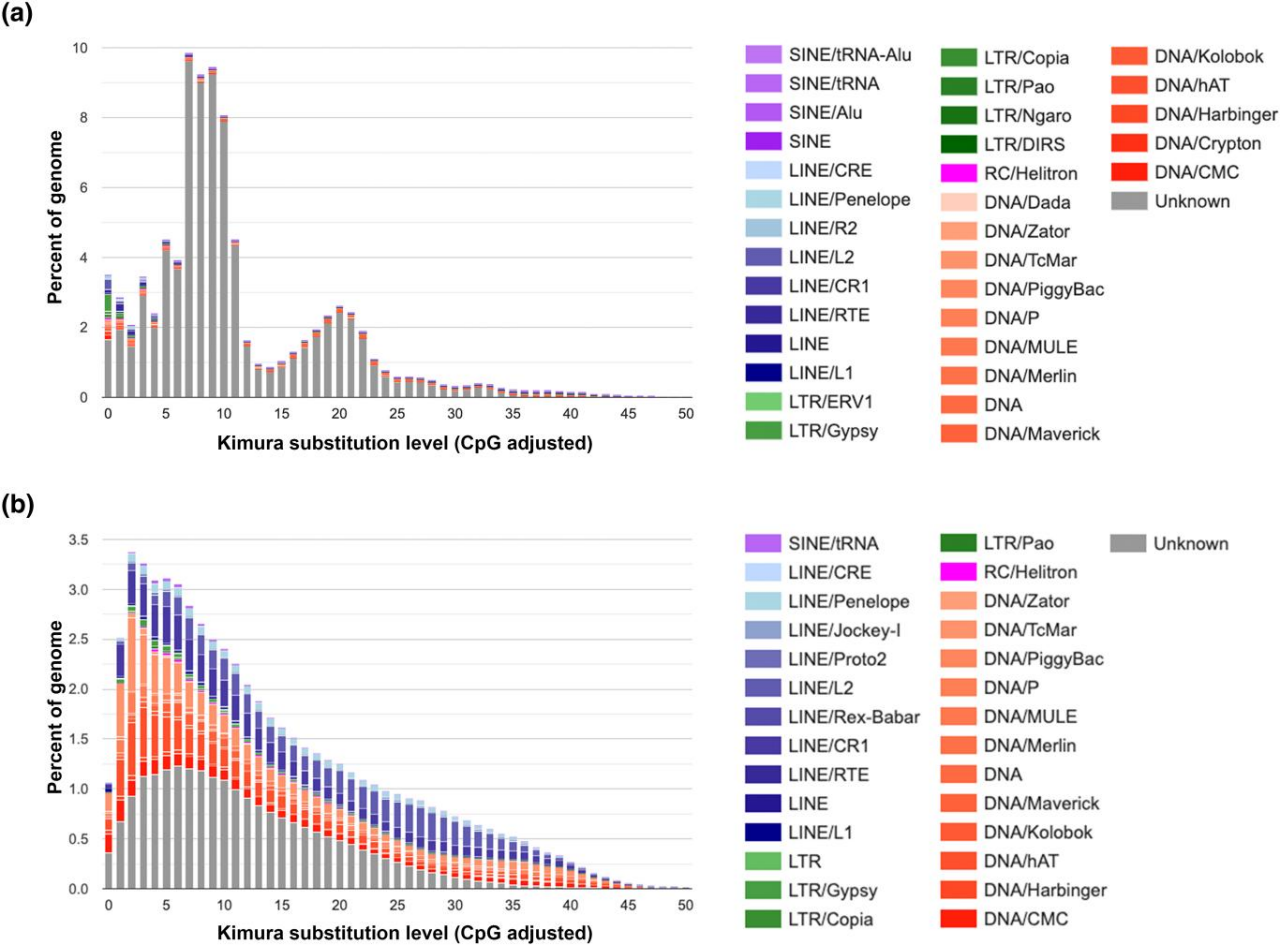
Interspersed repeat landscape of the *H. symbiolongicarpus* genome and *H. vulgaris* genome. a) Interspersed repeat landscape of *H. symbiolongicarpus*. b) Interspersed repeat landscape of *H. vulgaris*. The *x*-axis represents the level of Kimura substitution for repeat elements from the consensus sequences (relative age). The *y*-axis represents the relative abundance of each repeat family in the genome. Ancient active repeats are placed on the right side of the graph, and recently active repeats are on the left.

For the identification of repetitive sequences in the genome assembly, we generated a custom repeat library of our genome assembly using RepeatModeler. This library contains 2,300 repeat families. Then, RepeatMasker was run on the assembly using the custom library. Repetitive sequences accounted for 296 Mb (61%) of the HSymV2.0 assembly. The repetitive sequences are composed of total interspersed repeats (58.25%), simple repeats (0.70%), and others (Table 4). The total interspersed repeats can be further divided into retroelements (5.20%), DNA transposons (4.94%), rolling circles (0.55%), and unclassified elements (48.11%). Among the retroelements, LINEs (3.01%) were found to be the most abundant (Table 4). Among the DNA transposons, Tc1-IS630-Pogo (0.48%) and hobo-Activator (0.33%) were the top two elements (Table 4). These results are similar to the repeat analysis of the *H. vulgaris* genome. In the *H. symbiolongicarpus* genome, sequence divergence of the repeats revealed at least two discrete periods of repeat expansion (Fig. 3a). In contrast, in the *H. vulgaris* genome, more continuous repeat expansion was shown (Fig. 3b), which was also indicated by the previous study (Chapman *et al*. 2010).

**Table 4.** Summary of the RepeatMasker outputs.

		*H. symbiolongicarpus*			*H. vulgaris*	
	Number of elements	Length occupied (bp)	Percentage of sequence	Number of elements	Length occupied (bp)	Percentage of sequence
Retroelements	50,329	25,095,050	5.20	316,787	153,628,253	17.05
SINEs:	12,586	1,987,472	0.41	21,296	3,637,401	0.40
Penelope	9,072	2,726,592	0.56	52,322	17,817,731	1.98
LINEs:	27,013	14,541,672	3.01	281,818	140,096,759	15.55
CRE/SLACS	1,786	821,583	0.17	298	150,224	0.02
L2/CR1/Rex	9,860	6,205,894	1.29	207,793	111,582,339	12.39
R1/LOA/Jockey	0	0	0.00	659	166,379	0.02
R2/R4/NeSL	1,415	881,271	0.18	0	0	0.00
RTE/Bov-B	3,902	3,319,744	0.69	3,759	1,136,092	0.13
L1/CIN4	128	79,792	0.02	0	0	0.00
LTR elements:	10,730	8,565,906	1.77	13,673	9,894,093	1.10
BEL/Pao	1,500	1,411,183	0.29	2,320	2,078,705	0.23
Ty1/Copia	2,594	1,823,078	0.38	135	160,597	0.02
Gypsy/DIRS1	5,721	4,579,944	0.95	9,802	6,193,847	0.69
Retroviral	155	104,124	0.02	0	0	0.00
DNA transposons	56,331	23,868,445	4.94	536,767	243,675,039	27.05
hobo-Activator	3,454	1,578,931	0.33	183,730	81,989,900	9.10
Tc1-IS630-Pogo	11,142	2,305,486	0.48	128,135	56,913,582	6.32
En-Spm	0	0	0.00	0	0	0.00
PiggyBac	338	264,413	0.05	4,776	2,483,575	0.28
Tourist/Harbinger	5,311	1,339,873	0.28	6,623	1,752,620	0.19
Other	3,354	1,092,285	0.23	30,175	13,042,137	1.45
Rolling-circles	9,783	2,673,725	0.55	6,653	3,214,957	0.36
Unclassified:	410,631	232,360,741	48.11	764,256	184,468,711	20.48
Total interspersed repeats		281,324,236	58.25		581,772,003	64.57
Small RNA:	12,289	8,667,551	1.79	13,979	1,876,864	0.21
Satellites:	918	166,061	0.03	0	0	0.00
Simple repeats:	51,000	3,398,212	0.70	376,701	41,350,470	4.59
Low complexity:	10,920	531,858	0.11	49,800	2,588,884	0.29
Total bases masked:		295,609,498	61.21		630,803,178	70.02

### Gene prediction and functional annotation

**Fig. 4.**
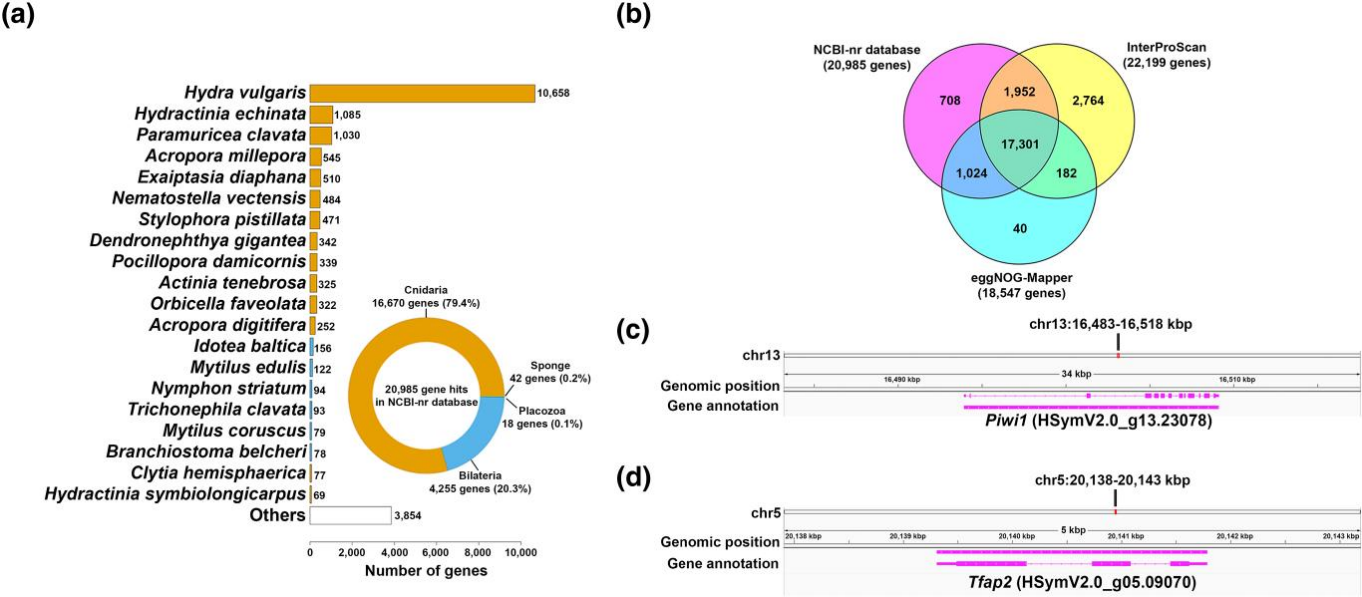
Gene annotation of the HSymV2.0 assembly. a) BLAST search results of the total 25,825 predicted protein-coding genes against the NCBI-nr database. Total 20,985 gene hits were identified in the NCBI-nr database. Top 20 species origins of the BLAST hits are shown as a bar plot. The inset pie chart represents the summary of species origins. The color scheme of the bar plot corresponds to that of the inset pie chart. b) Venn diagram showing functional annotations of the predicted protein-coding genes of the HSymV2.0 assembly. c) Gene structure of *Piwi1* on the chromosome 13. d) Gene structure of *Tfap2* on the chromosome 5.

In order to predict protein-coding genes in the HSymV2.0 assembly, we adopted a combination of ab initio prediction: homology-based prediction, and transcriptome-based prediction. For homology-based prediction, we used protein sequences from *H. vulgaris* and Hsym_primary_v1.0. For transcriptome-based prediction, publicly available RNA-seq reads were aligned to the assembly using the STAR aligner (Dobin *et al*. 2013). We then performed genome annotation using BRAKER2 (Brůna *et al*. 2021) with homology evidence or transcriptome evidence. Finally, the resulting genome annotations were integrated using TSEBRA (Gabriel *et al*. 2021) to produce the consensus gene model. We also mapped the gene coordinates of the gene annotation of the Hsym_primary_v1.0 assembly to the HSymV2.0 assembly using the Liftoff tool (Shumate and Salzberg 2020). Finally, we predicted a total of 25,825 protein-coding genes. Of the 22,022 protein-coding genes in the Hsym_primary_v1.0 assembly, 21,686 genes (98.5%) were mapped to 18,606 genes in the HSymV2.0 assembly (Supplementary Table 1). The predicted protein-coding genes showed a mean gene body length of 5,854 bp, a mean coding sequence (CDS) length of 1,346 bp, and a mean number of exons of 6.43. We detected 888 complete BUSCO genes out of a total of 954 BUSCO genes (93.1%) in the 25,825 predicted proteins. Among these genes, 813 appeared in single copies (85.2%), while 75 existed as duplicates (7.9%) (Table 5). These BUSCO scores were better than those of the predicted protein sequences of the Hsym_primary_v1.0 assembly (Table 5). Of all predicted protein-coding genes in our assembly, 20,985 protein-coding genes (81.3%) had significant homology to the sequences in the NCBI nonredundant database (nr) (Fig. 4a and Supplementary Table 2). Out of the total 20,985 genes that exhibited significant homology to genes in the NCBI-nr database, 16,670 genes (79.4%) were hit to Cnidaria genes (Fig. 4a). 4,255 genes (20.3%) were hit to Bilateria genes (Fig. 4a). The remaining 42 (0.2%) and 18 (0.1%) genes hit the Sponge and Placozoa genes, respectively. We identified that 22,199 protein-coding genes (86.0%) had protein domains using InterProScan (Supplementary Table 3) (Zdobnov and Apweiler 2001). We also used eggNOG-Mapper (Huerta-Cepas *et al*. 2019) for the predicted protein-coding genes and found that 18,547 genes were functionally annotated (Supplementary Table 4). Overall, 92.8% (23,971 genes) of all predicted protein-coding genes had functional annotations supported by one or more of the above databases (Fig. 4b). Stem cell factor genes are well predicted in our genome annotation. For example, *Piwi1* is a known marker for i-cells (Bradshaw *et al*. 2015; Gahan *et al*. 2016). In the HSymV2.0 assembly, *Piwi1* is on the plus strand of DNA in chromosome 13, spanning from base pair position 16,493,941 to 16,509,130 (Fig. 4c). Another example is transcription factor AP2 (*Tfap2*). This gene is a regulator of germ cell commitment (DuBuc *et al*. 2020). *Tfap2* is located on chromosome 5 from base pair position 20,139,307 to 20,141,788 on the plus strand of DNA (Fig. 4d).

**Table 5.** BUSCO scores from the predicted proteins.

	Current study (HSymV2.0)	Hsym_primary_v1.0
Complete BUSCOs (C)	888 (93.1%)	882 (92.5%)
Complete and single-copy BUSCOs (S)	813 (85.2%)	785 (82.3%)
Complete and duplicated BUSCOs (D)	75 (7.9%)	97 (10.2%)
Fragmented BUSCOs (F)	20 (2.1%)	12 (1.3%)
Missing BUSCOs (M)	46 (4.8%)	60 (6.2%)
Total BUSCO groups searched	954	954

### Synteny analysis between *H. symbiolongicarpus* and *H. vulgaris*

**Fig. 5.**
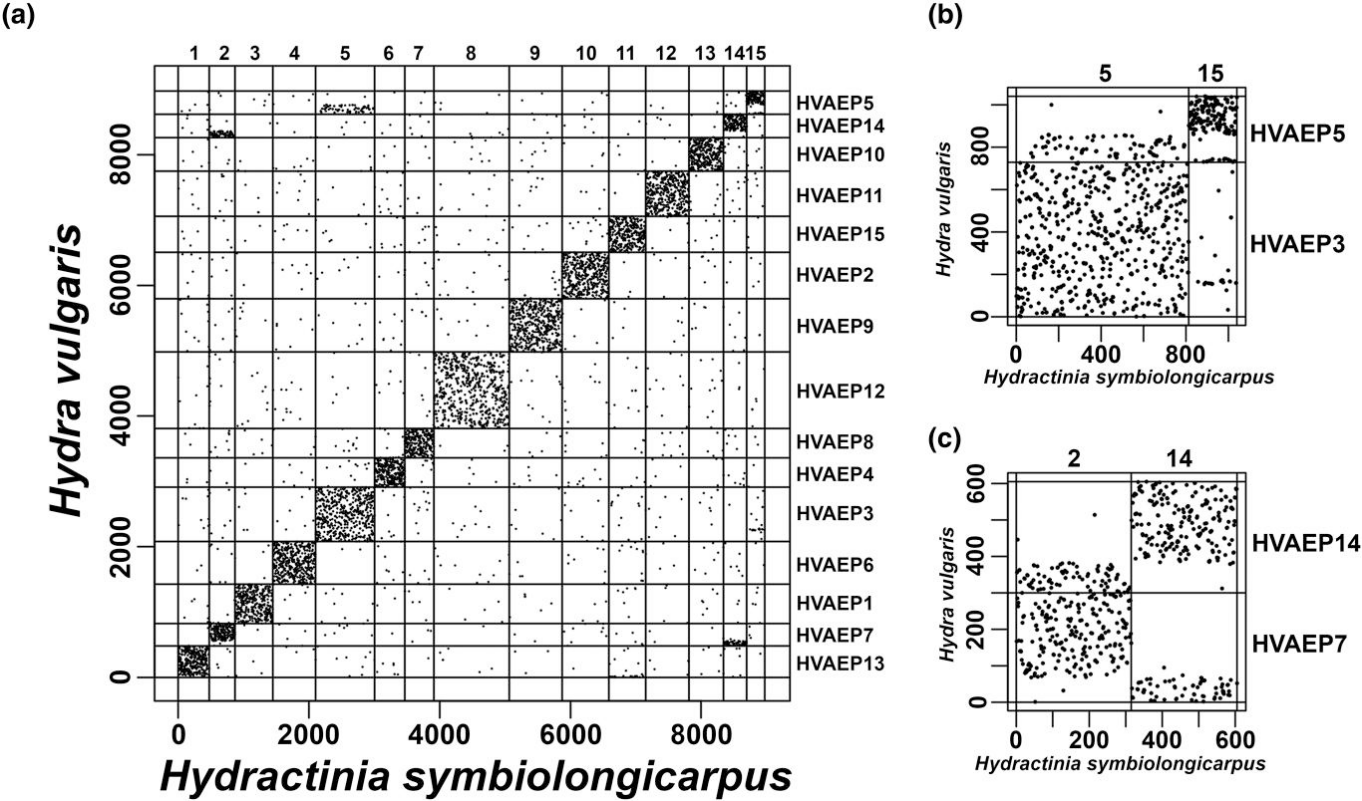
Oxford dot plot of orthologs between *H. symbiolongicarpus* and *H. vulgaris*. a) Oxford dot plot using the 8,974 orthologs. For both species, orthologs are sequentially numbered according to their genomic positions. Each dot represents the orthologs. b) Oxford dot plot using orthologs on scaffold 5 and 15 of *H. symbiolongicarpus* genome assembly and those on the HVAEP3 and HVAEP5 of the *H. vulgaris* genome assembly. c) Oxford dot plot using orthologs on the scaffold 2 and 14 of *H. symbiolongicarpus* genome assembly and those on the HVAEP7 and HVAEP14 of the *H. vulgaris* genome assembly.

We investigated the syntenic relationship between *H. symbiolongicarpus* and *H. vulgaris*. A total of 8,974 orthologs were identified (Supplementary Table 5). We generated the Oxford dot plot between *H. symbiolongicarpus* and *H. vulgaris* with these orthologs (Fig. 5a). Overall, this plot revealed that chromosome-scale synteny (macrosynteny) is highly conserved without collinearity between these species (Fig. 5a). Also, pairs of chromosomes (scaffold 5 and 15 of *H. symbiolongicarpus* vs HVAEP3 and HVAEP5 of the *H. vulgaris*, scaffold 2 and 14 of *H. symbiolongicarpus* vs HVAEP7 and HVAEP14 of the *H. vulgaris*) showed syntenic relationships suggesting the past interchromosomal translocations after the divergence between the species (Fig. 5b and c).

## Conclusion

In summary, we performed de novo genome assembly of *H. symbiolongicarpus* and obtained contig sequences with a size of 483 Mb and N50 of 28.8 Mb. The following Hi-C scaffolding resulted in 15 chromosome-level scaffolds containing 99.8% of the total genome assembly. This HSymV2.0 assembly is the first chromosome-level genome assembly of *H. symbiolongicarpus*. In the HSymV2.0 assembly, we predicted 25,825 protein-coding genes, and 92.8% of these genes (23,971 genes) were functionally annotated. The repeat and gene annotations of the genome assembly and comparative genome analysis between *H. symbiolongicarpus* and *H. vulgaris* highlight a unique genome evolution of *H. symbiolongicarpus*. Our novel chromosome-level genome assembly of *H. symbiolongicarpus* will be a significant resource for the biology of this organism. It also can be useful in comparative genomic studies in stem cell evolution in multicellular organisms.

## Supplementary Material

jkad107_Supplementary_Data

## Data Availability

The HSymV2.0 assembly, the gene annotation, and related files have been deposited at Figshare (https://doi.org/10.6084/m9.figshare.22126232.v1). The HSymV2.0 assembly has been also deposited at the NCBI under the accession JARBIS000000000. The PacBio HiFi reads are available at the Sequence Read Archive (SRA) under Accession Number SRR23493933. Hi-C reads have been deposited in the SRA with accession SRR23493932. Supplemental material available at G3 online.
