# Supplementary material for: Chromosome-level genome assembly of Hydractinia symbiolongicarpus
Source: G3 (Bethesda). 2023 May 18;13(8):jkad107. doi: 10.1093/g3journal/jkad107 (PMC10411563; doi:10.1093/g3journal/jkad107)
Supplement: jkad107_Supplementary_Data [file jkad107_supplementary_data.zip › Figure_S1_G3-2023-404160.pdf]

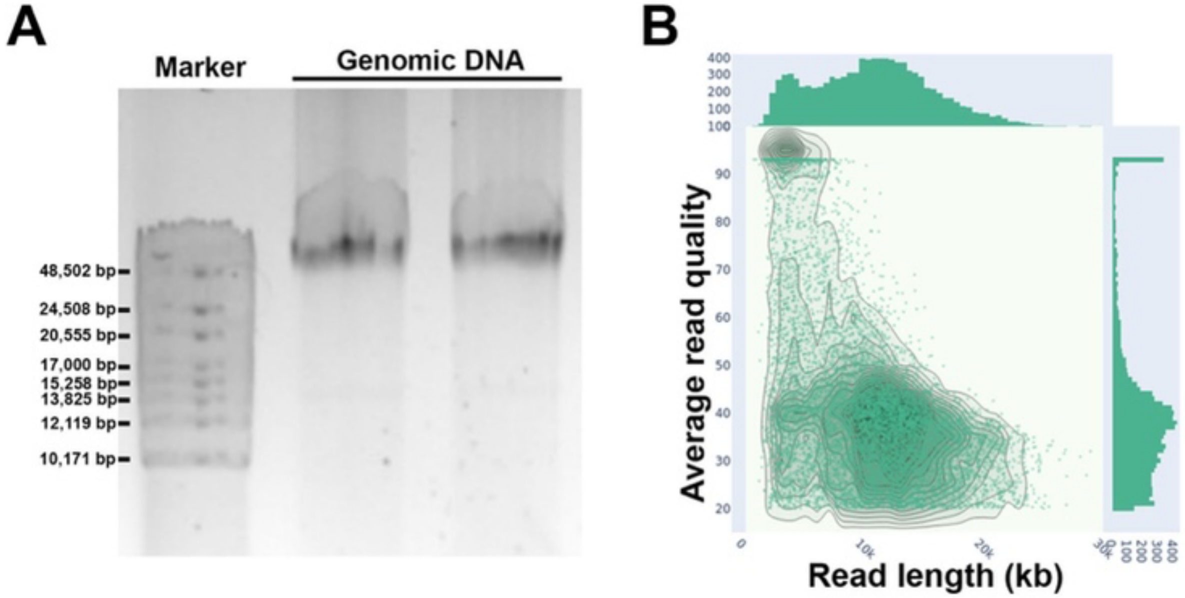


**Supplementary Figure 1.** PacBio HiFi long-read sequencing of the *H. symbiolongicarpus* genome. (A) Agarose gel electrophoresis of the extracted genomic DNA. (B) Distribution of read lengths and average read qualities of the PacBio HiFi long-reads. The solid lines are kernel density estimation.
