# Supplementary material for: Chromosome-level genome assembly of Hydractinia symbiolongicarpus
Source: G3 (Bethesda). 2023 May 18;13(8):jkad107. doi: 10.1093/g3journal/jkad107 (PMC10411563; doi:10.1093/g3journal/jkad107)
Supplement: jkad107_Supplementary_Data [file jkad107_supplementary_data.zip › Figure_S2_G3-2023-404160.pdf]

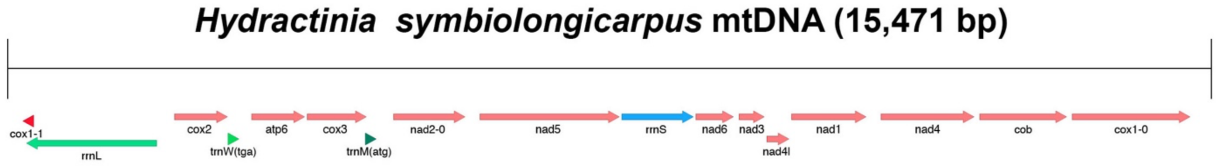

Supplementary Figure 2. Mitochondrial DNA of *H. symbiolongicarpus*. Arrows indicate gene orientations.
