# Supplementary material for: Chromosome-level genome assembly of Hydractinia symbiolongicarpus
Source: G3 (Bethesda). 2023 May 18;13(8):jkad107. doi: 10.1093/g3journal/jkad107 (PMC10411563; doi:10.1093/g3journal/jkad107)
Supplement: jkad107_Supplementary_Data [file jkad107_supplementary_data.zip › Figure_S3_G3-2023-404160.pdf]

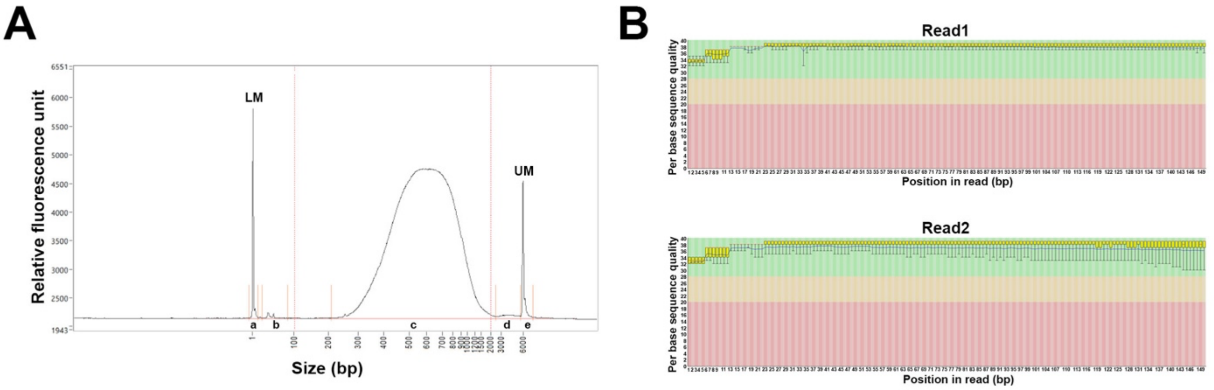

Supplementary Figure 3. Hi-C sequencing of the *H. symbiolongicarpus* genome. (A) Insert size distribution. The insert size distribution is approximately between 350 bp and 1,000 bp. The DNA concentrations of the DNA fragments in a (from 0 bp to 15 bp), b (from 25 bp to 84 bp), c (from 210 bp to 2,474 bp), d (from 2,474 bp to 5,698 bp), e (5,698 bp to 7,453 bp) are 0.0125 (ng/ μ L), 0.0198 (ng/ μ L), 4.27 (ng/ μ L), 0.0240 (ng/ μ L), 0.0058 (ng/ μ L), respectively. LM at 1 bp denotes the lower marker and UM at 6,000 bp denotes the upper marker. (B) Per base sequence quality of the Hi-C sequencing reads.
